# Electrospun Antibacterial Nanomaterials for Wound Dressings Applications

**DOI:** 10.3390/membranes11120908

**Published:** 2021-11-23

**Authors:** Aysegul Gul, Izabela Gallus, Akshat Tegginamath, Jiri Maryska, Fatma Yalcinkaya

**Affiliations:** 1 Institute for Nanomaterials, Advanced Technology and Innovation, Technical University of Liberec, Studentska 1402/2, 46117 Liberec, Czech Republic; aysegul.gul@tul.cz; 2 Faculty of Mechatronics, Informatics and Interdisciplinary Studies, Technical University of Liberec, Studentska 1402/2, 46117 Liberec, Czech Republic; izabela.gallus@tul.cz (I.G.); jiri.maryska@tul.cz (J.M.); 3 Faculty of Mechanical Engineering, Technical University of Liberec, Studentska 1402/2, 46117 Liberec, Czech Republic; akshattm93@gmail.com

**Keywords:** nanofiber, nanomaterial, wound dressing, antibacterial, tissue engineering, biomedical, electrospinning

## Abstract

Chronic wounds are caused by bacterial infections and create major healthcare discomforts; to overcome this issue, wound dressings with antibacterial properties are to be utilized. The requirements of antibacterial wound dressings cannot be fulfilled by traditional wound dressing materials. Hence, to improve and accelerate the process of wound healing, an antibacterial wound dressing is to be designed. Electrospun nanofibers offer a promising solution to the management of wound healing, and numerous options are available to load antibacterial compounds onto the nanofiber webs. This review gives us an overview of some recent advances of electrospun antibacterial nanomaterials used in wound dressings. First, we provide a brief overview of the electrospinning process of nanofibers in wound healing and later discuss electrospun fibers that have incorporated various antimicrobial agents to be used in wound dressings. In addition, we highlight the latest research and patents related to electrospun nanofibers in wound dressing. This review also aims to concentrate on the importance of nanofibers for wound dressing applications and discuss functionalized antibacterial nanofibers in wound dressing.

## 1. Introduction

The skin is the body’s largest organ, covering the entire external surface, which shields the internal organs from germs and thus aids in the prevention of infections. However, cuts, burns, surgical incisions, and illnesses such as diabetes can affect the structure and function of this organ.

Skin is divided into two layers, the epidermis and dermis. The epidermis is responsible for the healing process of the skin. A major part of the epidermal barrier is the stratum corneum, which plays an important role in this process. Several factors influence the health of the epidermal barrier, including the individual and the environment. The pH of the skin, the epidermal hydration, trans-epidermal water loss, and sebum excretion are the most important biophysical parameters that characterize the status of this barrier. In addition, the thickness of the epidermis’s outer layer, the size of corneocytes, and the composition of superficial lipids all impact the regenerative properties of the skin, which contributes to the various courses of dermatological diseases during the healing process [1,2,3]. The understanding of biophysical skin processes could be useful in the development of wound dressing materials to restore barrier functionality.

Wound dressings serve three functions: (a) Absorption of wound secretions, (b) protection of the wound from injury, and (c) protection of the wound from bacterial contamination [4]. There are high rates of morbidity and mortality associated with skin and soft tissue infections (SSTIs). Although some SSTIs can be successfully treated with medication, those that affect the subcutaneous tissue, fascia, or muscle can delay the healing process and lead to life-threatening conditions resulting from the delayed healing process. This necessitates the use of more effective treatments [5].

**Figure 1:**
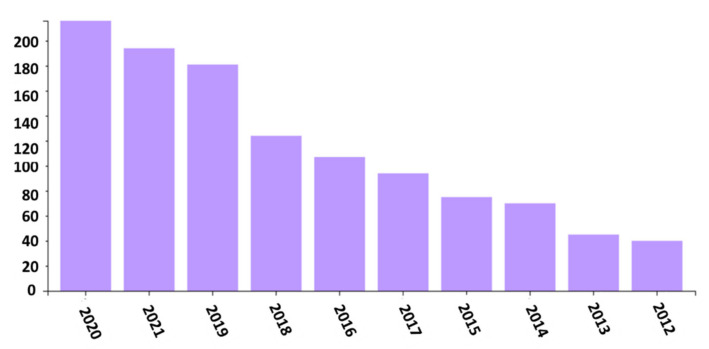
Recent publications related to electrospinning for wound dressing (September 2021).

Over the last few decades, a wide range of wound barrier materials have been studied, such as films, hydrogel, emulsions, composites, nano/microfibers, and so on [6,7,8,9,10,11,12]. Among them, nano/microfibers, in particular, have shown a promising future in wound dressing applications in recent years, making them very appealing to researchers. Figure 1 shows the growing number of publications in electrospinning for wound healing applications from the Web of Science database.

Nano-sized materials have a high surface area/volume ratio, facilitating efficient drug encapsulation and controlled release kinetics. Furthermore, the physicochemical properties of nanomaterials, such as hydrophobicity, surface charge, or particle size, can easily be modified and can be specifically designed to mimic the extracellular matrix (ECM) or other cellular components while avoiding natural clearance mechanisms such as the immune system [13,14,15]. The ECM is crucial in controlling cell behavior and regulates the cells and sends environmental signals to them for site-specific cellular regulation and distinguishes one tissue area from another [16]. In the early 1960s, researchers speculated that nanometer-sized features influence cell behavior [17]. According to recent studies, cells attach better to fibers that are smaller in diameter than the diameters of the cells [18,19]. Hence, it is critical to replicate the natural ECM size to create an ideal dressing that functions as a synthetic ECM to guide the wound healing process. The application of nanometer-sized fibers in wound dressings has been demonstrated over and over again of for its value in medical healing treatments.

It is critical to figure out how to create an in-vivo-like architecture that supports cell growth and re-creation as closely as possible. Due to the various parameters that can be controlled, the process of electrospinning is of paramount importance in the production of nanofibers. Using the process of electrospinning, nanofibrous wound dressing materials can be produced that have diameters ranging from a few nanometers to hundreds of nanometers, along with specified pore size, porosity, and patterns and alignments to meet various requirements.

Basic wound dressing properties include absorbency, bacterial barrier, oxygen permeability (gas transfer), non-adhesion to healing tissue, and bioactivity, all provided by electrospun nanofiber structures [20,21]. Abrigo et al. [22] gave an evolution of electrospun wound dressings. This classification is based on the previous commercial dressing classifications: Passive, interactive, advanced, and bioactive. Passive meshes in wound dressings provide physical (i.e., water and gas permeability) and morphological (i.e., adequate porosity and nanometer-scale) properties. Interactive electrospun meshes combine the necessary morphological and physical requirements for wound healing with the value-added capability to address optimal cell responses and limit bacterial proliferation in the wound bed. The primary strategy used to develop interactive systems is a combination of synthetic polymers and biopolymers with antibacterial properties and an affinity towards ECM components. Multicomponent systems are more similar to the ECM. Many researchers are currently developing drug-loaded nanofibrous meshes to manufacture interactive dressings capable of treating bacterial infection. The goal of bioactive electrospun meshes is to be a multifunctional system that combines various properties that are capable of treating all aspects of the wound. Adequate mechanical and physicochemical properties protect the wound, stimulate the healing process, and control the bacterial load in the wound bed [23].

Researchers are currently experimenting with various strategies to create electrospun meshes that can support wound healing while preventing infection. Table S1 in the supplementary information lists the most recent patents for wound dressing materials using electrospinning methods.

In this review, the main process associated with electrospinning are described, wound dressings which are currently available are presented; the advances in the fabrication of electrospun meshes as wound dressings are highlighted, focusing on the current strategies for developing effective antibacterial nanofibrous wound dressing. Compared to previous papers, this review highlighted the most recent, up to date literature about functional nanofibers and their application in the wound healing process. Furthermore, the recent achievements, developments and current challenges in antibacterial nanofiber webs for the purpose of wound dressings are discussed.

## 2. Electrospinning Process (Parameters and Biomedical Applications)

**Figure 2:**
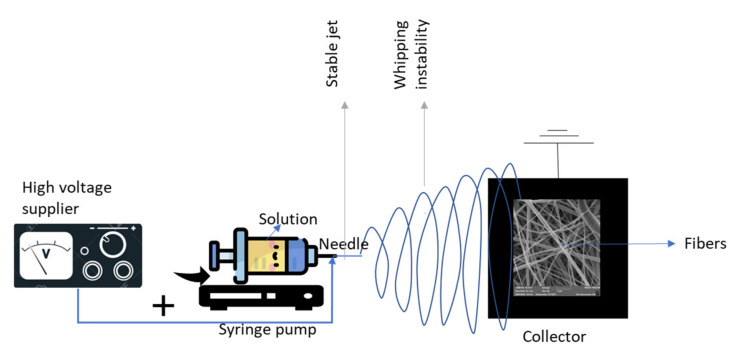
Electrospinning setup.

Electrospinning is a voltage-driven technique in which a liquid droplet is electrified to create a jet, which is then stretched and elongated to create fibers. The main setup for electrospinning, shown in Figure 2, includes a spinneret (syringe needle) connected to a high-voltage (5 to 50 kV) supplier, a syringe pump, and a grounded or oppositely charged collector.

The liquid is extruded from the spinneret during electrospinning, producing a pendant droplet due to surface tension. When a droplet is electrified, electrostatic repulsion between surface charges with the same sign deforms it into a conical shape known as the Taylor cone, from which a charged jet is released. As soon as the electric field reaches a critical value (where the repulsive electric forces overcome the surface tension forces), a charged solution jet is ejected from the tip of the Taylor cone. Because of bending instabilities, the jet initially extends in a straight line and subsequently undergoes severe whipping motions. An electric field can control the route of the jet as the jet is charged. As the jet flies in the air, the solvent evaporates, leaving behind a charged polymer fiber [23,24,25,26].

Certain factors have an impact on the electrospinning process. These factors are divided into three groups, as shown in Table 1. Researchers studied the effect of the controlling parameters, voltage, solution flow rate, concentration, molecular weight, distance, and solvent grade on the polymer jet’s electric current and charge density during electrospinning. The viscosity of the solution has been found to influence the fiber diameter linked to the polymer concentration and molecular weight. Increasing the solution viscosity has been linked to the formation of larger-diameter fibers [24,25]. Solution conductivity is also linked to the voltage and effect on fiber diameter; the high solution conductivity results from thin fibers [26,27]. The molecular weight is linked to viscosity, surface tension, and conductivity, which affects fiber diameter; if it is low, bead structures form [28,29]. The applied voltage is linked to the tip-to-collector distance, conductivity, and feed rate. Higher voltage results in thinner fibers, but jet instabilities occur if the voltage is too high, resulting in thicker fibers [30]. Temperature is linked to viscosity, and an increase in temperature results in a decrease in fiber diameter thanks to a decrease in viscosity [31].

**Figure 3:**
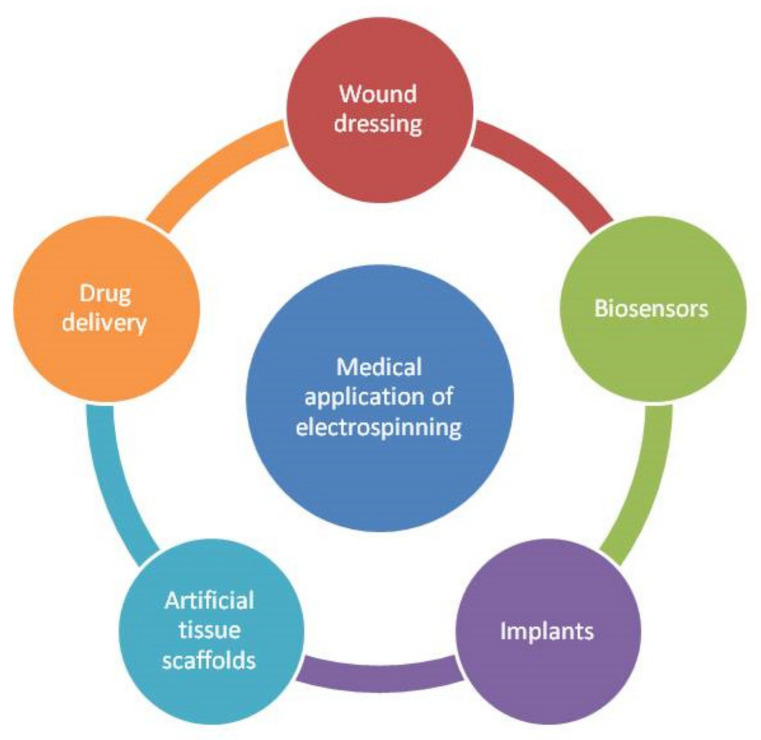
Medical applications of electrospinning.

Electrospun nanofibers are widely used in biomedical applications such as tissue-engineered scaffolds (vascular implants) [43], drug-delivery systems [44,45,46], and medical treatments in healthcare to improve wound healing [8,12,47]. Figure 3 shows the medical applications of electrospinning. Wound dressings are one of the most well-known of these applications. Thanks to the electrospinning technique, the fibers can be patterned or aligned to increase the contact efficiency of the cells. Furthermore, nanofibrous scaffolds have been shown to improve cell adhesion, protein adsorption, and cell growth and differentiation.

Antibacterial nanofibers have received special attention. With the incorporation of antimicrobial agents, the design goal of wound dressing materials has been to avoid or reduce infection, which is the cause of bacteria. Antimicrobial nanofibrous wound dressings have recently emerged as a viable technique to decrease infection and wound bacterial colonization to improve the healing process (Table S2 shows recent studies for antibacterial electrospun wound dressings).

## 3. Antibacterial Nanofibers for Wound Dressing

One of the major causes of chronic infections can be linked to bacterial infections [48,49,50,51], which fester at a very high rate in existing wounds; thus, the need to use antibacterial materials is of paramount importance. With a large surface area, antibacterial nanofibers allow for the efficient integration of antibacterial agents [52]. In recent years, nanotechnology has advanced at a blistering pace. The areas of research under nanotechnology are also expanding at an exponential rate. One of the research areas under this revolution is nanomedicine, and over recent decades, this field has shown great potential of becoming a major field of research. Research in this field has led to drastic improvement of human health [53]. Several techniques have been utilized to produce nanofibers, such as melt spinning, chemical vapor deposition, sinter technology, solution spinning, and electrospinning. Among these techniques, the electrospinning technique has been determined as the most cost-effective method in producing continuous nanofibers from numerous polymers or compounds [54,55,56,57,58]. The nanofibers produced via electrospinning have a large specific area, a high porosity, and huge interest in applications in tissue engineering, regenerative medicine, and wound dressing.

The foremost function of the skin is to protect the internal organs, muscles, and bones, which can be affected by burns, cuts, or illnesses. The process of healing a wound starts instantly when the skin is affected. The presence of bacteria will reduce the efficacy of healing the wound and increase the chances for an infection to occur and fester. The absence of Gram-positive organisms such as staphylococcus aureus and streptococcus pyogenes would exponentially increase the wound’s healing rate. Thus, these microorganisms must be eliminated quickly. After surgery or an injury, the exposed tissues may be in danger of contracting an infection, which may lead to diseases, and in severe cases, it may even lead to death [48]. Thus, the dressing of the wounds would help prevent infections and maintain an environment conducive to healing wounds [59]. For wound healing, electrospun nanofibers have the following features that are imperative for their usage:

- Mimicry of the composition.
- Mimicry of the structure.
- Incorporation of bioactive materials.
- Mechanical mimicry.
- Regulation of the skin cell response [48].

### 3.1. Mimicry of the Composition

Various materials have been used in the field of wound healing, such as hydrogels, gas-foaming formed scaffolds, or decellularized porcine dermal matrices [60,61,62]. However, these materials cannot reproduce the skin’s extracellular matrix (ECM) [63]. Electrospinning has found traction in recent years for wound healing, as it can be used to produce biomimetic nanofibers with the required features from numerous synthetic and natural polymers [64]. Collagens, laminins, elastins, proteoglycans, and polysaccharides are some of the proteins present in the ECM of skin [65]. Due to electrospinning’s multifaceted nature, nanofibers of type I and III, which make up a major portion of the dermal matrix, can be produced [66]. By direct electrospinning, surface immobilization, or blending, electrospun nanofibers can be produced that have a high degree of similarities with the ECM of the skin. Table 2 shows the various electrospun nanofibers that can be utilized to recreate the ECM of the skin.

### 3.2. Mimicry of the Structure

Upon observation under an electron microscope [88], human skin was found to have three zones (papillary, mid, and deep zones), which are composed of a fine layer of fibers near the epidermis with a thick layer of fiber bundles and a loosely arranged fiber bundle layer. The fiber bundles consist of parallelly aligned fibrils. It was later found that the collagen present in the skin has a basket-weave structure [89,90,91]. To achieve this structure, numerous attempts were made to produce electrospun nanofibers similar to it [92,93,94]; using the weaving techniques present in the industry, forays have been made to produce nanofiber yarns with a basket-weave [95,96]. Using a process called ‘noobing’, 3D nanofiber scaffolds with a basket-weave structure were produced [97].

### 3.3. Incorporation of Bioactive Materials

With the introduction of therapeutic agents, the process of wound healing can be accelerated at the site of a wound. The local delivery of therapeutic agents such as antioxidants, anesthetics, enzymes, growth factors, and antimicrobial agents can be comprehensively achieved with the help of electrospun nanofibers [98]. The advantage of using electrospun nanofibers to deliver these agents over the commonly used drug delivery system is that the nanofibers have a fast response rate with greater control over the release rate [99,100]. The therapeutic agents can be introduced into the electrospun nanofibers via co-axial electrospinning or emulsion electrospinning [101,102]. The process of CO2 impregnation or infusion or surface immobilization can be utilized to introduce the therapeutic agents into the electrospun nanofibers [103,104]. Table S3 in Supplementary Materials lists therapeutic agents that can be incorporated with electrospun nanofibers.

### 3.4. Mechanical Mimicry

The parameters of the scaffold can influence the process of tissue regeneration, and cellular behavior used [105]. Thus, the mechanical properties of late have come into the limelight [106]. Due to a low degree of orientation and extension of polymer chains, electrospun nanofibers have low tensile strength and Young’s modulus [107]. Thus, it is of preponderant importance to select the appropriate raw material that can encompass the desired properties [108]. Surface coating, mechanical treatments, and thermal treatments can be utilized to introduce the required properties into the electrospun nanofibers [109,110,111]. In Table 3, a collection of nanofibers that come close to the mechanical properties of the human skin and a comparison with the mechanical properties is presented.

### 3.5. Regulation of the Skin Cell Response

For a wound to heal ECM deposition, skin cell proliferation and migration must take place. It was found that a spreading morphology was shown by cells when electrospun nanofibers with type I collagen, laminin, and integrin ligands were used for wound dressings [67]. In a study conducted by Yoo et al., it was found that the mRNA levels for loricin and keratin 1 were higher when the PCL nanofibers were cultured with keratinocytes and chemically conjugated human epidermal growth factors were utilized [115]. To increase re-epithelialization when scaffolds are used, aligned PVA nanofibers can be used, as they would assist the keratinocytes in the wound healing process [116].

## 4. Biopolymeric Nanofibrous Antibacterial Wound Dressings

The ecofriendly nature and biocompatibility of biopolymers are some of the characteristics due to which biopolymers are extensively studied to create wound dressings with the desired characteristics. Since the biopolymers show a high degree of similarity to the ECM structure, high bioactivity, and are biodegradable, polysaccharide biopolymers, among the many biopolymers utilized, are comprehensively studied. The following are the various biopolymers used in the study of wound healing:

- Collagen.
- Alginate.
- Chitosan.
- Gelatin [117].
- Fibronectin and fibrin [118].

Collagen is used in wound dressing due to the following reasons:

- Low antigenicity and inherent biocompatibility.
- Increase in fibroblast production and permeation.
- Helps to preserve leukocytes, macrophages, fibroblasts, and epithelial cells.
- Attracts fibroblasts and encourages the deposition of new collagen to the wound bed.

Collagen nanofiber webs are similar to native tissue architecture and are easily remodeled due to their simple structure, easy preparation, availability, and relative uniformity. Collagen nanofiber helps the healing process but does not show anti-bacterial properties. An antibacterial additive or treatment is needed. On the other hand, chitosan not only shows biocompatibility and biofunctionality but also antibacterial, analgesic, antioxidant, and neuroprotective properties. Electrospun chitosan nanofiber webs are promising candidates for wound healing.

Gelatin nanofibers are interesting for use in the wound healing process due to their biodegradable, easy to spin, controllable thickness, and physical stability properties. Gelatin nanofiber does not show antibacterial properties. However, mixing with antibacterial materials such as chitosan, curcumin, or nanoparticles can improve the antibacterial property of gelatin nanofibers [119,120,121].

To treat burn injuries, a cellulose nanofibril wood-based wound dressing has been developed. Cellulose is a very commonly available polysaccharide that helps speed up wound healing by providing assistance in the processes of epithelialization, granulation, and tissue regeneration [122]. Cellulose can be obtained from bacteria (Acetobacter xylinum) and plants. The cellulose obtained from the bacteria is called bacterial cellulose; this cellulose has great mechanical characteristics, biocompatibility, biodegradability, and physicochemical properties required to produce a wound dressing material [123]. Bacterial cellulose can be used to regenerate blood vessels, reconstruct the damaged tissues, and wound healing since it can mimic the structure of the ECM with great ease and similarities [124]. The cellulose-based wound dressing properties can be elevated by introducing antimicrobial drugs, hormones, antioxidants, and enzymes [125]. Gallic acid can be used to functionalize cellulose acetate nanofibers, as it is a polyphenol compound with antioxidant, anti-inflammatory, and antibacterial characteristics [126]. The ECM of vertebrates contains Hyaluronic acid (HA), a naturally occurring nonimmunogenic linear polysaccharide [127]. For wound healing, numerous hydrogels based on HA were examined. The HA was functionalized with thiol [128], glycidyl methacrylate [129], and DNA [130], to help with networking. The HA used in wound dressing materials mostly supports cellular migration, proliferation, and absorbing exudates, hence, leading to the regeneration of tissues and healing of the wound [131]. Shell fibers of HA core-poly (lactic-co-glycolic acid, PLGA) with epigallocatechin-3-0-gallate (EGCG) were produced and developed by Shin et al. [100] and used as a wound dressing on diabetic rats, and it was found that the HA/PLGA-E fibers used helped to increase the rate of the wound healing process. A blend of HA/poly (vinyl alcohol) (PVA) nanofibers was also developed for wound dressing [132]; here, the HA is carried by the PVA polymer along with the addition of hydroxypropyl-βcyclodextrin (HP βCD), which is used a stabilizing agent in electrospinning, to allow a water-based fabrication process. Due to a high degree of biocompatibility and biodegradability, chitosan (CS) and chitin are good options in developing wound dressing materials, with chitin being one of the most available natural amino polysaccharides whose production is equal to that of cellulose and can be found in fungi cell walls as well as the exoskeletons of crustaceans, insects, and invertebrates. For the purpose of wound dressing, PVA/CS/tetracycline hydrochloride (TCH) [133], honey/PVA/CS [134], and bacterial cellulose/CS/polyethylene oxide (PEO) CS-based antibacterial nanofibers have been suggested. With very low toxicity levels, good biocompatibility, and inexpensive cost, alginate, an anionic polymer derived naturally [132], can be utilized to produce wound dressings made from collagen alginate, gelatin alginate calcium alginate, and calcium sodium alginate [125]. When used for wound healing, alginate maintains appropriate levels of moisture and greatly reduces bacterial activity at the wound site and accelerates the wound’s healing process [135]. It is blended with various synthetic polymers to produce an electrospun nanofibrous wound dressing based on alginate [136]. Collagen nanofibers were used to produce wound dressing materials by Zhou et al. [137], which were used to vitalize epidermal differentiation and human keratinocytes and increase the rate of healing of the wound. The collagen fibrils were paired with synthetic and natural polymers, which would help maintain the moisture and help absorb the exudate from the wound and accelerate the process of wound healing [138]. Yao et al. [139] developed a gelatin/keratin blended nanofiber wound dressing material, which enhanced the migration, adhesion, and cell proliferation leading to vascularization and healing of the wound, which was observed in the animal test model.

Natural biopolymers such as silk fibroin (SF) obtained from the mulberry silkworm, Bombyx mori, are utilized in biomedical applications due to their inexpensiveness, biocompatibility, biodegradability, green processing, and very low inflammatory response [140]. Along with these properties, SF has great exudate absorption capacity, pliability, and adherence. This can be used as a stand-alone or combined with alginate, multiwalled carbon nanotubes, chitosan, etc. [125]. The skin’s environment can be mimicked to a high degree by the SF, leading to an accelerated wound healing process and minimized scarring [141]. Thus, wound dressing materials based on SF are being researched and developed [142]. Antioxidant Fenugreek/SF nanofiber wound dressing material was fabricated by Selvaraj and Fathima [143], which, along with wound healing characteristics, also helps with collagen deposition and complete re-epithelialization. The potential for wound healing using biopolymeric nanofibers is excellent, but the properties offered are seldom enough to fulfill both disinfection and wound healing. Functional agents that help in accelerating the wound healing rates must be used, hence hybridizing the biopolymeric nanofibers [118].

## 5. Nanoparticle Containing Nanocomposite Antibacterial Nanofibers

The wound healing process is continually put on the line and tested with the presence of bacteria. When bacteria are present, they may lead to inflammation of the wound and delay the process of healing. Bioactive wound dressings are a new field of wound dressing and show great potential in displacing the conventional wound dressing methods [118]. Wound dressing materials can be modified with surface-functionalized agents, bio blends, and antibacterial nanocomposites or nanoparticles to have antibacterial action. In recent years, silver nanoparticles were used in polymeric nanofibers due to their ability to resist bacterial activity [144]. The wound dressing material physically shields the wound from bacterial activities and helps with the differentiation of fibroblasts and their migration at the wound site. According to the mode of loading and the type of antibacterial agent utilized, various types of wound dressings are present such as hydrogels, films, foams, or sponges [118].

An open wound is open for bacterial attacks, increasing inflammation and leading to long periods of wound healing. As a result, it would lead to impeding the production of new granulation tissues and damage the ECM’s constituents. When an antimicrobial dressing is applied at the site of the wound, pathogens cannot enter the wound as their pathway is blocked, and those that entered prior to applying the wound dressing will be eliminated efficiently. Moreover, the immune system is induced to promote the migration of keratinocytes/fibroblasts, leading to faster wound healing [145].

Nanoparticles such as zinc oxide, silver, iron oxide, and gold are used for biodetection, medical devices, drug delivery, and wound healing [146]. Because of their ability to fight human pathogens, they can be used to design wound dressings. For this reason, metallic nanoparticles have recently attracted much interest from researchers. Silver nanoparticles are of particular interest. They have strong toxicity and a large surface area, increasing contact with pathogens [147]. Silver nanoparticles and silver complexes have already found wide use in producing antimicrobial materials and wound healing [6]. The incorporation of metal nanoparticles and metal oxide into the polymeric membrane structure is considered one of the better solutions for developing dressings with antimicrobial properties. Materials such as hydrogels, nanocomposites, and nanofibers have high porosity, excellent gas permeability, and a high surface-area-to-volume ratio. These are required in wound healing as they ensure proper cellular respiration, hemostasis, exudate removal, improved skin regeneration, and hydration [148]. In the design of wound healing materials, it is believed that the best strategy is to combine various non-conventional antimicrobial formulations in order to harness their synergistic effects to overcome microbial resistance [146]. Combining hydrogels, nanocomposites, or nanofibers with nanoparticles seems to be the optimal solution for creating wound healing materials. The introduction of Ag nanoparticles into the polymer structure can be carried out by different methods such as electrospinning, chemical modification, or hydrogel formation [149,150]. Hongli et al. managed to obtain porous silver nanoparticle/chitosan composites with wound healing activity by in situ reductions of silver nanoparticles with gelatin [6]. Kumar et al. created a chitin hydrogel/nano ZnO composite bandage [148], while Jatoi et al. obtained poly(vinyl alcohol) composite nanofibers embedded with silver-anchored silica nanoparticles [150]. Table 4 summarizes various research studies using nanocomposites, nanofibers, hydrogels, and nanoparticles to produce materials suitable for wound healing.

## 6. Biofunctionalized Antibacterial Nanofibers

Biofunctionalized antibacterial nanofibers are a type of wound dressing material where the biopolymeric nanofibers are surface functionalized with amino acids and antimicrobial peptides [118]. The two most important biopolymers are chitosan and silk fibroin when dealing with biofunctionalized nanomaterials, since they allow various antimicrobial agents to be attached via the numerous functional groups present. The antimicrobial peptides (AMPs) bound to the surface of the nanofibers are studied widely [164,165,166]. Due to the biocompatibility offered by the AMPs, they have now become one of the most utilized antimicrobial additives for wound dressings. This wound dressing system produced is a hybrid system, and the type of AMP tailors the antimicrobial activity of these hybrid systems utilized [118]. For the AMP to be immobilized on the surface of the nanofiber, numerous approaches are utilized. Co-spinning and covalent binding are approaches implemented in producing the nanofibers with AMP immobilized onto the surface [166]. The process of covalent immobilization provides the best process, as this leads to negligible leaching of the AMP and long-term stability and nontoxicity [167]. Various antibacterial biohybrid nanofibrous wound dressings are produced based on the surface functionality of silk fibroin (SF). On the SF nanofibers, various functional groups such as carboxyl, hydroxyl, phenol, and amines are loaded [168]. It has been observed that SF biohybrid nanofibers do not allow the growth of bacteria [118]. If the immobilized factor amount is higher, the antibacterial activity is higher. Over a period of three weeks, the effect of biofunctionalized nanofibers remains constant, disregarding the temperature of storage. It has been found that the bacteria S. Aureus can reduce the efficiency of AMPs by lowering the negative surface charge, changing the fluidity of their membrane, or using their pumps to keep the AMPs away [169].

Another biopolymer that is biocompatible and biodegradable is chitosan, and this has excellent antimicrobial properties against various microorganisms such as fungi, algae, viruses, and bacteria [170,171,172,173,174]. The electrostatic interactions of the amine groups present in chitosan undergo electrostatic interactions on the cell wall [172]; due to this, the permeability of the cell wall is altered. Hence, the osmotic balance is disrupted, which leads to the restriction of the growth of the microorganism. In addition, the leakage of intracellular electrolytes occurs due to the hydrolysis of peptidoglycans [175]. Due to this, several blends of functionalized chitosan nanofibers have been suggested [118]. The positive charge of the amino acids is the key factor in the protection against microorganisms; to this end, L-asparagine [176], L-arginine [177], and L-lysine [118] have been grafted onto the nanofibers of chitosan to increase the density of positive charge present. A wound dressing made from deacetylated/arginine functionalized chitosan has been developed [178]. The bio functional component helps with the higher deposition ability of collagen; this, in turn, helps with the healing of the wound at a greater rate [118]. Table 5 below shows examples of biofunctionalized antibacterial materials.

Proteins can be combined with polymer structures using electrospinning. However, this is a very challenging process due to their molecular weight, the ionic, hydrogen, and disulfide bonds present, and the complexity of their structure. During the electrospinning of proteins, the most important factor is the proper choice of solvent. One must consider their solubility in a given solvent and the degree of unfolding and entanglement of the protein chain. In addition, the solvent affects the fiber size, crystallinity, morphology, and mechanical properties of the protein. Therefore, adding a synthetic polymer during electrospinning is necessary for this to occur continuously and without interference. The production of wound healing dressings during electrospinning uses animal or plant-based proteins [185,186,187,188]. The activity, degradation, and stability of the material are determined by the proteins’ size, chemical structure, purification process, and protein isolation. The purity and composition of the obtained raw material affect the reproducibility of the electrospinning process and the properties of the final product [47,188].

Silver-based compounds have been used since the early 1970s for wound care applications [189] and hence the combination of silver with sulphadiazine was established, which led to the usage of silver in wound dressings [190]. The active antimicrobial entity in wound dressings that makes use of silver is the silver ion, and these ions react with the thiol (-SH) groups, leading to the generation of reactive oxygen species (ROS), and this is the major contributor to the antibacterial efficacy of the wound dressing used. Silver ions, when released, have the potential to cross various biological partitions [189].

Due to the history of the usage of silver in therapeutic agents, the potential toxicity of silver is a well-documented fact. The ingestion or dermal exposure or inhalation of salts of silver in sufficient amounts lead to Argyria and Argyrosis, which is blue–grey discoloration of the skin and eyes. This occurs mainly due to the deposition of the silver precipitates. Although argyria is not toxic in nature, it leads to disfigurement and, hence, this is considered an undesirable effect [191,192]. Historical studies have shown that high dosages of silver nitrate lead to gastrointestinal damage and rarely lead to fatalities [191]. There has been little to no evidence to support the fact that silver in any form might be toxic in nature to the cardiovascular or immune, reproductive, or nervous systems in humans [193,194]. A threshold limit value of 0.01 mg/m^3^ for metallic silver in soluble form and 0.1 mg/m^3^ for metallic silver has been set by the American Conference of Governmental Industrial Hygienists (ACGIH). These values have been set based on the limit values for protection against Argyria [195].

Liu et al. [196] conducted a cytotoxicity study of the nanofibrous membranes produced from PEU and CA for 3 days in in vitro conditions with rat skin fibroblast cells according to DS/EN ISO10993-5 [197]. The results of these tests showed that the pure PEU and co-spun PEU/CA nanofibers containing PHMB had no toxicity towards the fibroblast cells of the rats, as the cells showed adhesion to the nanofibrous membranes and showed growth. Thus, this led to the conclusion that the polymers used were biocompatible and safe to use as wound dressing materials.

To determine the biocompatibility of nanofiber-based wound dressing materials, clinical trials have to be undertaken to gain extensive knowledge, but the number of studies being undertaken at the clinical phase is very limited [198]. The number of clinical trials to determine the effects of electrospun nanofibers can be found on the clinical trial website [199].

## 7. Conclusions

The interest in electrospun nanofiber mats has risen drastically due to their unique properties such as high specific surface area, highly porous structure, tight pore size and pore size distribution, interconnected pores, and good chemical and biological activity.

Herein, we have briefly reviewed the role of the nanofiber web in wound dressing applications. For an ideal wound dressing, a future perspective, the requirements are:

- Nontoxic to mammal cells.
- Nonantigenic.
- Good mechanical resistance.
- Elastic and flexible.
- Antibacterial.
- Permeable for gas exchange.
- Inexpensive.
- Long shelf-life.

Incorporating functional nanoparticles or bioactive agents into nanofibers improves the antibacterial property of wound dressing materials. There is no doubt that the nanofiber web has provided a promising wound dressing material in biomedical applications for its unique properties. In recent years, the limitation of low production behind the electrospinning process has been due to industrial production devices. On the other hand, bringing nanofiber webs into the clinical field still needs to be improved. With more clinical research and improved functional nanofiber web, the electrospun nanomaterials can offer an unprecedented breakthrough in biomedical applications.

## Figures and Tables

**Table 1:** Effecting parameters of electrospinning.

Parameters	Effect on Fibers	References
Solution Parameters		
Viscosity	A higher viscosity results in a large fiber diameter. If the viscosity is very low, there will be no continuous fiber formation; if the viscosity is too high, the jet will be difficult to eject from the needle tip.	[24,32,33]
Solution Concentration	A minimum solution concentration is required for fiber formation in the electrospinning process. Increased concentration leads to larger diameters.	[34]
Molecular weight	Low molecular weight solutions tend to form beads rather than fibers, whereas high molecular weight nanofiber solutions produce fibers with a larger average diameter.	[25]
Solution electrical conductivity	When the electrical conductivity of the solution increases, the diameter of the electrospun nanofibers decreases significantly. Beads may also be observed due to the solution’s low conductivity, which results in insufficient elongation of a jet by electrical force to produce uniform fiber.	[27,35]
Surface tension	The surface tension of the solution can drive droplets, beads, and fibers and the solution’s low surface tension ensures that spinning occurs with a lower electric field requirement.	[36,37,38]
Process Parameters		
Applied voltage	It has been discovered that increasing the electrostatic potential leads to thinner fibers. However, if too much voltage is applied, the jet may become unstable, and the fiber diameters may increase.	[39]
Distance from needle to the collector	The traveling time of the polymeric jet is affected. Traveling time should be long enough for complete evaporation of the solvent.	[40,41]
Volume feed rate	Increasing the feed rate resulted in an increase in fiber diameter and the formation of a bead structure.	[36,37]
Environmental Parameters		
Humidity	High humidity can cause pores on the surface of the fiber.	[33,42]
Temperature	Temperature increases cause a decrease in fiber diameter due to a decrease in viscosity.	[42]

**Table 2:** Electrospun nanofibers mimicking the ECM of the skin.

Composition	Approximate Diameter	Reference
Collagen	460 nm	[66,67]
Collagen/chitosan	(134 ± 42) nm	[68]
Collagen/PCL	(170 ± 0.075) nm	[69]
Collagen/Zein	(423–910) nm	[70]
Collagen/elastin/PEO	(220–600) nm	[71]
Laminin I	(90–300) nm	[72]
PCL/gelatin	(470 ± 120) nm; (409 ± 88) nm	[73,74]
Gelatin	(570 ± 10) nm	[75]
Polyurethane/gelatin	(0.4–2.1) μm	[76,77]
HA/PEO	(70–110) nm	[78]
Silk fibroin/chitosan	(185.5–249.7) nm	[48]
Silk fibroin/PEO	(414 ± 73) nm; 1 μm	[79,80]
Chitin	163 nm	[81]
Carboxyethyl chitosan/PVA	(131–456) nm	[82]
Chitosan/gelatin	(120–220) nm	[83]
PLGA	(150–225) nm	[84]
PLGA/collagen	(170–650) nm	[85]
Chitosan/PEO	(130–150) nm	[86]
Hyperbranched polyglycerol	(58–80) nm	[87]

**Table 3:** Nanofibers mimic the mechanical properties of the human skin with a comparison.

	Human Skin	PCL/Collagen	HA/PLGA	PLGA/Collagen
**Tensile modulus (MPa)**	15–150	21.42 ± 0.04	28.0	40.43 ± 3.53
**Ultimate tensile stress** **(MPa)**	1–32	8.63 ± 1.44	1.52	1.22 ± 0.12
**Ultimate tensile strain** **(%)**	35–115	24.0 ± 7.16	60.07	96 ± 13
**Reference**	[112]	[113]	[114]	[112]

**Table 4:** An overview of recent wound dressing materials constructed from nanoparticles and nanomaterials.

Material	Nanoparticles	Bacterial Species	Ref.
Carboxymethyl Chitosan/Polyethylene Oxide Nanofibers (CMCTS–PEO)	Ag (12–18 nm)	*S. aureus, P. aeruginosa, E. coli, fungus Candida albicans*	[149]
Alginate/Nicotinamide Nanocomposites	Ag (20–80 nm)	*S. aureus* and *E. coli*	[150]
Nanofibrous Poly vinyl alcohol, chitosan	Ag	*S. aureus* and *E. coli.*	[151]
Nanofibrous mats from cellulose acetate	Ag	*S. aureus* and *E. coli.*	[152]
Nanofibrous membrane from Gum Arabic, polycaprolactone, polyvinyl alcohol	Ag	*S. aureus, E. coli, P. aeruginosa* and *C. albicans*	[153]
PVA-co-PE nanofibrous membrane	Ag	*S. aureus* and *E. coli.*	[154]
Electrospun peppermint oil on polyethylene oxide/Graphene oxide	CeO_2_	*S. aureus* and *E. coli.*	[155]
Hyaluronic acid	ZnO	*S. aureus, B. subtilis, E. coli, P. aeruginosa,* and *V. cholerae*	[156]
Chitosan/cellulose acetate	CeO_2_	*S. aureus* and *E. coli.*	[157]
Chitosan/poly(N-vinylpyrrolidone)	TiO_2_	*E. coli, S. aureus, B. subtilis* and *P. aeruginosa*	[158]
Chitosan/pectin	TiO_2_	*E. coli, S. aereus, A. niger, B. subtilis, P. aeruginosa*	[159]
Electrospun Chitosan/Gelatin	Fe_3_O_4_	*S. aureus* and *E. coli.*	[160]
Β-Chitin Hydrogel	Ag (4–8 nm)	*S. aureus* and *E. coli.*	[161]
Chitosan/Polyvinyl Alcohol Hydrogel, Collagen	Ag (4–19 nm)	*P. aeruginosa* and *S. aureus*	[162]
Linseed hydrogel	Ag (10–35 nm)	*E. coli, S. mutans, A. niger, S. epidermidis, P. aeruginosa, S. aureus, acillus subtilis, Actinomyces odontolyticus*	[163]

**Table 5:** Biofuntionalized antibacterial materials with proteins.

Protein	Co-Polymer	Antimicrobial Agent	Bacterial Species	Ref.
Zein	PU	Ag NPs	*E. coli, S. aureus*	[151]
Zein	PU/CA	Streptomycin	*V. vulnificus, S. aureus,* *B. subtilis*	[177]
Keratin	PVA, PEO	Ag NPs	*E. coli, S. aureus*	[178]
Collagen	CS	ZnO	*S. aureus, E. coli*	[179]
α-lactoglobulin	PEO	Ampicillin	*E. coli, P. aeruginosa,* *B. thailandensis*	[180]
Silk fibroin	PEO	TiO_2_ NPs	*E. coli*	[181]
Silk fibroin	-	Ag NP coating	*S. aureus, P. aeruginosa*	[182]
Silk fibroin	PEO	Cu_2_O NPs	*S. aureus, E. coli*	[47]
Lactoferrin	Gelatin	-	*E. coli, S. aureus*	[183]
Gelatin	Alginatedialdehyde	Ciprofloxacin, gentamicin	*P. aeruginosa, S.* *epidermidis*	[184]

## Data Availability

Not applicable.

## Supplementary Materials

The following are available online at https://www.mdpi.com/article/10.3390/membranes11120908/s1, Table S1: Patents from 2020 to 2021 for antibacterial electrospun wound dressings materials, Table S2: Studies for antibacterial electrospun wound dressing materials in 2020–2021, and Table S3. Therapeutic agents used in nanofibers.
