# Supplementary material for: Electrospun Antibacterial Nanomaterials for Wound Dressings Applications
Source: Membranes (Basel). 2021 Nov 23;11(12):908. doi: 10.3390/membranes11120908 (PMC8707140; doi:10.3390/membranes11120908)
Supplement: Supplementary file 1 [file membranes-11-00908-s001.zip › membranes-1458378-supplementary.pdf]

membranes

MDPI

Review

Electrospun Antibacterial Nanomaterials for Wound Dressings Applications

Aysegul Gul [1], Izabela Gallus [2], Akshat Tegginamath [3], Jiri Maryska [2], Fatma Yalcinkaya [2,*]

[1] Institute for Nanomaterials, Advanced Technology and Innovation, Technical University of Liberec, Studentska 1402/2, 46117, Czech Republic ; aysegul.gul@tul.cz
[2] Faculty of Mechatronics, Informatics and Interdisciplinary Studies, Technical University of Liberec, Studentska 1402/2, 46117, Czech Republic; izabela.gallus@tul.cz (I.G.); jiri.maryska@tul.cz (J.M.)
[3] Faculty of Mechanical Engineering, Technical University of Liberec, Studentska 1402/2, 46117, Czech Republic; akshattm93@gmail.com
* Correspondence: fatma.yalcinkaya@tul.cz; Tel.: +420-485353389

Abstract: Chronic wounds are caused by bacterial infection and create major healthcare discomfort. Hence, an antibacterial material is needed to use in wound dressing. Traditional wound dressing materials are unable to meet the needs for antibacterial properties. For this reason, designing an antibacterial wound dressing is demanded to accelerate the healing period. Electrospun nanofibers offer a promising solution to the management of wound healing. Nanofibers provide wide options for loading antibacterial compounds into the web. This review gives us an overview of some recent advances of electrospun antibacterial nanomaterials in wound dressing. First, we provide a brief overview of the electrospinning process, nanofibers in wound healing and then discuss electrospun fibers by incorporating various antimicrobial agents used in wound dressings. In addition, we highlight the latest research and patents related to electrospun nanofibers in wound dressing. This review aims to concentrate on the importance of nanofibers for wound dressing applications and discuss functionalized antibacterial nanofibers in wound dressing.

Keywords: nanofiber; nanomaterial; wound dressing; antibacterial; tissue engineering; biomedical; electrospinning.

SUPPLEMENTARY INFORMATION

Citation: Gul, A.; Gallus, I.; Tegginamath, A.; Maryska, J.; Yalcinkaya, F. Electrospun Antibacterial Nanomaterials for Wound Dressings Applications. *Membranes* **2021**, *11*, 908. https://doi.org/10.3390/membranes11120908

Academic Editor: Andrea Ehrmann

Received: 26 October 2021
Accepted: 19 November 2021
Published: 23 November 2021

Publisher's Note: MDPI stays neutral with regard to jurisdictional claims in published maps and institu-tional affiliations.

Copyright: © 2021 by the authors. Licensee MDPI, Basel, Switzerland. This arti-cle is an open access article distrib-uted under the terms and conditions of the Creative Commons Attribution (CC BY) license (https://creativecommons.org/licenses/by/4.0/).

Table S1. Patents from 2020 to 2021 for antibacterial electrospun wound dressings materials.

Title	Publication number	Publication date	References
Electrospun Double-Layer Long-Acting Antibacterial Medical Dressing and Preparation Method Thereof	CN111012941A	2020-04-17	[1]
Antibacterial Disinfection Nanofiber Medical Dressing	CN111557790A	2020-08-21	[2]
Polyvinyl Alcohol-Nano-Silver Dressing, Preparation Method and Application Thereof	CN111321514A	2020-06-23	[3]
Preparation Method of Antibacterial Electrostatic Spinning Fiber Based on Polyion Liquid, Antibacterial Electrostatic Spinning Fiber and Application Thereof	CN111636110A	2020-09-08	[4]
Composite Dressing with Antibacterial Function and Preparation Method Thereof	CN112169006A	2021-01-05	[5]
Plant Extract-Containing Wound Dressing and Application Thereof	CN112316196A	2021-02-05	[6]
Preparation Method of Antibacterial PBC/PLA/TP Composite Dressing	CN112831917A	2021-05-25	[7]
Polyethylene-Vinyl Alcohol/Gelatin Composite Electrostatic Spinning Wound Dressing and Preparation Method Thereof	CN112741926A	2021-05-04	[8]
Preparation Method of Antibacterial PLA/PBC/CS Composite Dressing	CN112831918A	2021-05-25	[9]

Antibacterial Elastic Composite Medical Dressing and Preparation Method Thereof	CN112755236A	2021-05-07	[10]
Alginate Composite Dressing and Preparation Method of Composite Dressing	CN112206342A	2021-01-12	[11]
Functional Graphene-Based Fiber Hygienic Material	CN111420112A	2020-07-17	[12]
Medical Composite Nanofiber Dressing as Well as Preparation Method and Application Thereof	CN111939307A	2020-11-17	[35]
Electrostatic Spinning Membrane Capable of Releasing Nitric Oxide Based on Near-Infrared Response as Well as Preparation Method and Application of Electrostatic Spinning Membrane	CN111945301A	2020-11-17	[13]
Multi-Component Double-Layer Composite Nano-Film Dressing and Production Method Thereof	CN112569397A	2021-03-30	[14]
Medical Composite Dressing for Wound Repair and Preparation Method Thereof	CN111529748A<br>CN111529748B	2020-08-14<br>2021-06-04	[37]
Composite Hydrogel Wound Dressing and Preparation Method Thereof	CN111518288A	2020-08-11	[15]
Polyvinyl Alcohol/Carboxymethyl Chitosan Nanofiber Medical Dressing as Well as Preparation Method and Application Thereof	CN111118734A<br>CN111118734B	2020-05-08<br>2021-06-01	[16]
Antibacterial Healing-Promoting Nanofibre Scaffold and Nanofibre Scaffold Patch Made by Scaffold	CN111701070A	2020-09-25	[17]

Preparation Method of Cellulose Diacetate-Based Three-Dimensional Scaffold with Both Antibacterial Property and Biocompatibility	CN112064193A	2020-12-11	[18]
High-Air-Permeability Degradable Drug-Loaded Skin Wound Dressing and Preparation Method Thereof	CN112807475A	2021-05-18	[19]
Chitosan Cross-Linked Antibacterial Nanofiber Membrane and Preparation Method Thereof	CN111334934A	2020-06-26	[20]
Self-Assembled Nanofiber Dressing for Promoting Vascularization Repair of Diabetes Ulcer, And Preparation Method and Application Thereof	CN111588901A	2020-08-28	[43]
Method for Preparing Antibacterial Gelatin Film Through Electrostatic Spinning	CN112481711A	2021-03-12	[21]
Preparation and Application of Hadscs-Loaded Double-Layer Skin Bionic Hydrogel Composite Scaffold	CN112675360A	2021-04-20	[22]
Wound Protection Film for Preventing Pneumonia Virus Infection for Pediatrics and Preparation Method Thereof	CN111265709A	2020-06-12	[23]
Photo-Crosslinking/Electrostatic Spinning Preparation and Application of Hydrogel Composite Scaffold with Double-Layer Skin Structure	CN112569399A	2021-03-30	[24]
Skin Wound Repair Plaster Loaded with Cell Regulatory Factor and Preparation Method of Skin Wound Repair Plaster	CN112336908A	2021-02-09	[25]
Drug Sustained and Controlled Release Platform System with Dual Nano Composite Structure	CN112353780A	2021-02-12	[48]
Multifunctional Intelligent Composite Gel Material as Well as Preparation Method and Application Thereof	CN111073196A	2020-04-28	[26]

Multifunctional Medical Material and Preparation Method and Application Thereof	CN112546295A	2021-03-26	[27]

Table S.2. Studies for antibacterial electrospun wound dressing materials in 2020-2021.

Polymer	Antibacterial/other agents	Type of bacteria	Type of Electrospinning	Electrospinning parameters	Highlights	References
Poly (vinyl alcohol) (PVA)	Honey and curcumin longa (turmeric) extrac	Staphylococcus aureus	Needle electrospinning	12.3 kV, 23 kV, 0.45 kW a flow rate of 1.5 mL/h, 65% relative humidity and at 27oC, distance of collector 15 cm	Because of the presence of antibacterial constituents in honey and turmeric extract, the developed samples formed inhibition zones of 29 mm and 38 mm, respectively, whereas no inhibition zone was formed for the same bacteria when PVA nanofiber was used alone.	[28]
PVA	Palmarosa oil and phytoncide oil	Staphylococcus aureus and Candida albicans	Emulsion (single-nozzle elec-trospinning)	Feed rate; from 0.2 to 1.8 ml/h. Voltage: 21-25 kV. The needle gauges: 23 (0.33 mm inner diameter.) and 27 (0.20 mm inner diameter . The tip-to-collector distance :18 cm	The palmarosa oil-containing electrospun core/sheath structured PVA nanofibrous membranes have a high potential as bioactive wound-dressing materials.	[29]
Polycaprolactone (PCL)/PVA_Pectin (PEC)	Chelidonium majusL. (C. majus)	Staphylococcus aureus (S. aureus) and Pseudomonas aeruginosa (P. aeruginosa)	Needleless emulsion electrospinning	Voltage: 80 kV, distance from collector: 15 cm, Electrode rotation rate: 55 Hz	PCL/PVA-PEC nanofiber meshes have been found to have the potential to be used to prevent bacterial wound infection and consequently accelerate wound healing.	[30]
PCL	Achyranthes aspera (AS) and Datura metel (DM) leaf	-	Needle electrospinning	High-voltage DC power supply (14 kV), needle gauge: 18-mm, feed rate: 1.4 ml/h, distance from	PCL-AS and PCL-DM nanofiber mats were found to have the ability to form mepidermis and granular tissue at an earlier stage of wound healing,	[31]

				collector; 12.5 cm.	according to histo-pathological evaluation results.	
PVA/beta-Cydo-dextrin (PVA/beta-CD)	Silver nanoparticles (Ag NPs), and riboflavin (RF)	Staphylococcus aureus and Escherichia coif	Needle electrospinning	Applied voltage:20 kV, Feed rate: 0.6 mL/h, rotation speed: 1200 rpm, syringe; 21 gauge:	It was concluded that Ag nanoparticles and RF implanted scaffolds could be an effective wound dressing material.	[32]
Poly(L-lactide) (PLLA)	Ofloxacin (OFLX)	Staphylococcus aureus and Escherichia coli	Needle electrospinning	Voltage: 20 kV Feed rate: 0.4 mL/h Distance from collector: 10 cm	In vitro cell viability tests revealed that PLLA Nanofiber mats loaded with OFLX up to 5% w/w were viable. It has been demonstrated that it is biocompatible and capable of cell proliferation.	[33]
Thermoplastic polyurethane (TPU), antibacterial N-halamine incorporated polymer (AP)	N-Halamine	Gram positive S. aureus (ATCC6538), Gram negative E. coli (ATCC8739) and fungi involved with Saccharomyces cerevisiae (ATCC9763) and Aspergillus niger (ATCC16404)	Needle electrospinning	Voltage: 1.5 kV/cm Feed rate: 1.0 mL/h Distance from collector: 10 cm	It has been discovered that NMs can release Cl+ in a sustained manner for approximately 6 days, following the Weibull pattern, indicating the desired stability. It was also determined that the NMs can be recharged using a simple chlorination process using diluted sodium hypochlorite solution, with a recharge efficiency of approximately 80% when compared to the original one.	[34]
PVA/chitosan	Kaolin	Staphylococcus aureus, Pseudomonas aeruginosa	Needle electrospinning	Voltage:15-18kV Feed rate: 0.5ml/h Distance from collector: 15 cm	After 14 days, rats treated with kaolin-containing mats were approximately 97.62 percent +/- percent, compared to PVA/chitosan and sterile gauze, which were 86.15 percent +/- 8.11 percent and 78.50 percent +/-, respectively. It demonstrated a significant wound closure of up to 4.81 and 4.22 percent,	[35]

					respectively. Histopathological studies revealed that the PVA/chitosan/kaolin group formed dense and regular collagen fibers, whereas wounds treated with sterile gauze or PVA/chitosan scaffolds formed random and loose collagen fibers.	
PCL/gelatin	B12 vitamine	-	Needle electrospinning	Voltage:20 kV Feed rate: 1.0ml/h Distance from collector: 15 cm Mandrel speed: 550 and 600 rpm	The study results showed that after 14 days, vitamin B12-containing dressing could significantly improve wound closure compared to vitamin B12-free scaffolds (92.27 +/- 6.84 percent vs. 64.62 +/- 2.96 percent).	[36]
Poly(epsilon-caprolactone) (PCL), quaternized chitosan-graft-polyaniline (QCSP)	QCSP	Staphylococcus aureus (ATCC 29213) and Escherichia coli (ATCC 8379)	Needle electrospinning	Voltage: 17 kV and −4 kV Feed rate: 0,04.0ml/-1 Distance from collector: 15 cm Gauge: 19	PCL/QCSP15 (15 wt% QCSP in the sample) demonstrated a good balance of antibacterial activity and cell proliferation, indicating that it significantly accelerated wound healing in a mouse full-thickness wound defect model compared to commercial dressing (TegadermTM Film). and a nanofibrous membrane made entirely of PCL (PCL/QCSP0).	[37]
Polylactic acid glycolic acid (PLGA)/silk fibroin (SF)	Silk fibroin (SF), artemisinin (ART)	-	Needle electrospinning	Voltage: −1 kV, –20 kV Feed rate: 0.8 mL/h Distance from collector: 15 cm Gauge: 23 Mandrel speed: 600 rpm	In this study, it has been proven that the produced PLGA/SF/ART2 fibrous membranes have a good anti-inflammatory effect.	[38]
PLA	Zinc oxide (ZnO) nanoparticles,	Staphylococcus aureus (Gram-	Needle electrospinning	Voltage: 20 kV, flow rate:0.6 mL.h-	Antibacterial studies against Gram-positive Staphylococcus aureus and Gram-negative	[39]

	tranex-amic acid (TXA)	positive, ATCC 25023) and Escherichia coli (Gram-negative, PTCC 1399)		1, tip-to-collector distance: 10 cm, and collector speed: 100 RPM	Escherichia coli showed a 98% reduction in colony forming units and a 75% reduction in colony forming units, respectively. In vivo studies in the rat model revealed that nanocomposite dressing containing PLA/ZnO/TXA nanofibers aided wound healing.	
PU, PAN, SPA	Polyhexamethylene guanidine hydrochloride (PHGC)	Staphylococcus aureus and Escherichia coli	Needle electrospinning	Voltage: 20 kV, flow rate: 0.1 mL/min., tip-to-collector distance: 18 cm	The designed tri-layered dressing exhibited approximately 100 percent antibacterial ability against Staphylococcus aureus and Escherichia coli when the concentration of antibacterial agents polyhexamethylene guanidine hydrochloride (PHGC) was 0.06 wt percent in the study.	[40]

Table S.3. Therapeutic agents used in nanofibers.

**Purpose**	**Nanofiber**	**Therapeutic agent used**	**Reference**
Antibacterial	Chitosan/PVA	Lysozyme	[41]
Antibacterial	Gelatin/polyurethane; gelatin; polyurethane; poly(ethylene-co-vinyl alcohol)	Silver	[42–45]
Antibacterial	PCL; alginate/PVA	ZnO	[46]
Antibacterial	PLGA	Cefoxitin sodium	[47]
Antibacterial	Chitosan	Gentamicin	[48]
Antibacterial	Polyurethane/dextran; PVA/poly(vinyl acetate)	Ciprofloxacin HCl	[49]
Antibacterial	Cellulose acetate/polyester urethane	Polyhexamethylene biguanide	[50]
Pain management and antibacterial	PLLA	Lidocaine, mupirocin	[51]
Hemostasis	PLLA	Fibrinogen	[42]
Antioxidant	PCL	Curcumin	[52]
Angiogenesis	Chitosan/PEO; HA/collagen	VEGF	[53]
Angiogenesis, granulation tissue	Polyurethane; HA/collagen	PDGF-BB	[54]

formation			
Keratinocytes migration and maturation, angiogenesis	PCL–PEG/PCL; poly(l-lactic acid)-co-poly-(ε-caprolactone); HA/collagen; PCL/PEG	EGF	[55]
Cell adhesion, proliferation, ECM secretion, re-epithelialization and skin appendages regeneration, angiogenesis	PELA; HA/collagen	Basic-FGF	[56]

References

1. He, J.; Qi, L.; Ou, K.; Zhou, Y.; Yu, W.; Liu, C.; Yuan, P.; Liao, X.; Zhang, Y.; Chen, Y.; et al. Electrospun Double-Layer Long-Acting Antibacterial Medical Dressing and Preparation Method Thereof 2020.
2. Chen, L. Antibacterial Disinfection Nanofiber Medical Dressing 2020.
3. Gao, J.; He, Y.; Hao, Y.; Shen, M.; Li, J.; Zhao, Y.; Ran, Y.; Wang, S.; Zhao, X.; Ran, X.; et al. Polyvinyl Alcohol-Nano-Silver Dressing, Preparation Method and Application Thereof 2020.
4. Guo, J.; Yan, F.; Wang, M.; Sun, Z. Preparation Method of Antibacterial Electrostatic Spinning Fiber Based on Polyion Liquid, Antibacterial Electrostatic Spinning Fiber and Application Thereof 2020.
5. He, L.; Wan, J. Composite Dressing with Antibacterial Function and Preparation Method Thereof 2021.
6. Feng, B.; Su, D.; Li, H.; Shi, J. Plant Extract-Containing Wound Dressing and Application Thereof 2021.
7. Gu, X.; Zhang, D.; Liu, S.; Chen, H. Preparation Method of Antibacterial PBC/PLA/TP Composite Dressing 2021.
8. Guo, D.; Li, T.; Sun, J. Polyethylene-Vinyl Alcohol/Gelatin Composite Electrostatic Spinning Wound Dressing and Preparation Method Thereof 2021.
9. Gu, X.; Zhang, D.; Liu, S.; Chen, H. Preparation Method of Antibacterial PLA/PBC/CS Composite Dressing 2021.
10. Wu, D.; Feng, Q.; Li, M.; Wei, A.; Liu, C.; Xu, H.; Tao, Y.; Zhou, T.; Zhao, L. Antibacterial Elastic Composite Medical Dressing and Preparation Method Thereof 2021.
11. He, L.; Wan, J. Alginate Composite Dressing and Preparation Method of Composite Dressing 2021.
12. Sun, J. Functional Graphene-Based Fiber Hygienic Material 2020.
13. Ding, D.; Yan, F.; Zhang, W.; Cheng, N.; Wang, W. Electrostatic Spinning Membrane Capable of Releasing Nitric Oxide Based on Near-Infrared Response as Well as Preparation Method and Application of Electrostatic Spinning Membrane 2020.
14. Fei, Y. Multi-Component Double-Layer Composite Nano-Film Dressing and Production Method Thereof 2021.
15. Zheng, Y.; Feng, X.; Shi, X.; Yang, J. Composite Hydrogel Wound Dressing and Preparation Method Thereof 2020.
16. Cheng, J.; Huang, Z.; Zhang, J. Polyvinyl Alcohol/Carboxymethyl Chitosan Nanofiber Medical Dressing as Well as Preparation Method and Application Thereof 2020.
17. Yan, T.; Qin, C.; Liu, Y.; Xin, Q. Antibacterial Healing-Promoting Nanofibre Scaffold and Nanofibre Scaffold Patch Made by Scaffold 2020.
18. Lang, M.; Liang, W. Preparation Method of Cellulose Diacetate-Based Three-Dimensional Scaffold with Both Antibacterial Property and Biocompatibility 2020.
19. Chen, N. High-Air-Permeability Degradable Drug-Loaded Skin Wound Dressing and Preparation Method Thereof 2021.
20. Ge, Y.; Tang, J.; Fu, Y.; Xie, Y.; Chen, P.; Xie, H. Chitosan Cross-Linked Antibacterial Nanofiber Membrane and Preparation Method Thereof 2020.
21. Yu, N.; Li, J.; Ma, F.; Yang, P.; Zhu, Z.; Li, G.; Hou, W. Method for Preparing Antibacterial Gelatin Film through Electrostatic Spinning 2021.
22. Li, Y.; Zang, H.; Song, T. Preparation and Application of HADSCs-Loaded Double-Layer Skin Bionic Hydrogel Composite Scaffold 2021.
23. Chen, C. Wound Protection Film for Preventing Pneumonia Virus Infection for Pediatrics and Preparation Method Thereof 2020.
24. Li, Y.; Zang, H.; Song, T. Photo-Crosslinking/Electrostatic Spinning Preparation and Application of Hydrogel Composite Scaffold with Double-Layer Skin Structure 2021.
25. Yuan, B.; He, X.; Qiu, Y. Skin Wound Repair Plaster Loaded with Cell Regulatory Factor and Preparation Method of Skin Wound Repair Plaster 2021.

26. Yang, L.; Lin, X.; Mao, Y.; Bai, Y.; Chen, T.; Li, P. Multifunctional Intelligent Composite Gel Material as Well as Preparation Method and Application Thereof 2020.
27. Zou, J.; Li, L.; Li, J. Multifunctional Medical Material and Preparation Method and Application Thereof 2021.
28. Shahid, M.A.; Ali, A.; Uddin, M.N.; Miah, S.; Islam, S.M.; Mohebbullah, M.; Jamal, M.S.I. Antibacterial Wound Dressing Electrospun Nanofibrous Material from Polyvinyl Alcohol, Honey and Curcumin Longa Extract. J. Ind. Text. 2021, 51, 455–469, doi:10.1177/1528083720904379.
29. Lee, K.; Lee, S. Electrospun Nanofibrous Membranes with Essential Oils for Wound Dressing Applications. Fiber. Polym. 2020, 21, 999–1012, doi:10.1007/s12221-020-9300-6.
30. Mouro, C.; Gomes, A.P.; Ahonen, M.; Fangueiro, R.; Gouveia, I.C. Chelidoniummajus L. Incorporated Emulsion Electrospun PCL/PVA_PEC Nanofibrous Meshes for Antibacterial Wound Dressing Applications. Nanomaterials 2021, 11, 1785, doi:10.3390/nano11071785.
31. Suryamathi, M.; Viswanathamurthi, P.; Seedevi, P. Herbal Plant Leaf Extracts Immobilized PCL Nanofibrous Mats as Skin-Inspired Anti-Infection Wound Healing Material. Regen. Eng. Transl. Med., doi:10.1007/s40883-020-00193-9.
32. Balakrishnan, S.B.; Thambusamy, S. Preparation of Silver Nanoparticles and Riboflavin Embedded Electrospun Polymer Nanofibrous Scaffolds for in Vivo Wound Dressing Application. Process Biochem. 2020, 88, 148–158, doi:10.1016/j.procbio.2019.09.033.
33. Rade, P.P.; Garnaik, B. Ofloxacin-Loaded PLLA Nanofibrous Mats for Wound Dressing Applications. ACS Appl. Bio Mater. 2020, 3, 6648–6660, doi:10.1021/acsabm.0c00290.
34. Luo, H.; Yin, X.; Tan, P.; Shi, Y.; Gu, Y.; Zeng, R.; Tan, L. Engineering an Antibacterial Nanofibrous Membrane Containing N-Halamine for Recyclable Wound Dressing Application. Mater. Today Commun. 2020, 23, 100898, doi:10.1016/j.mtcomm.2020.100898.
35. Salehi, M.; Farzamfar, S.; Ehterami, A.; Paknejad, Z.; Bastami, F.; Shirian, S.; Vahedi, H.; Koehkonan, G.S.; Goodarzi, A. Kaolin-Loaded Chitosan/Polyvinyl Alcohol Electrospun Scaffold as a Wound Dressing Material: In Vitro and in Vivo Studies. J. Wound Care 2020, 29, 270–280, doi:10.12968/jowc.2020.29.5.270.
36. Farzanfar, S.; Kouzekonan, G.S.; Mirjani, R.; Shekarchi, B. Vitamin B12-Loaded Polycaprolacton/Gelatin Nanofibrous Scaffold as Potential Wound Care Material. Biomed. Eng. Lett. 2020, 10, 547–554, doi:10.1007/s13534-020-00165-6.
37. He, J.; Liang, Y.; Shi, M.; Guo, B. Anti-Oxidant Electroactive and Antibacterial Nanofibrous Wound Dressings Based on Poly(Epsilon-Caprolactone)/Quaternized Chitosan-Graft-Polyaniline for Full-Thickness Skin Wound Healing. Chem. Eng. J. 2020, 385, 123464, doi:10.1016/j.cej.2019.123464.
38. Peng, Y.; Ma, Y.; Bao, Y.; Liu, Z.; Chen, L.; Dai, F.; Li, Z. Electrospun PLGA/SF/Artemisinin Composite Nanofibrous Membranes for Wound Dressing. International Journal of Biological Macromolecules 2021, 183, 68–78, doi:10.1016/j.ijbiomac.2021.04.021.
39. Molapour Rashedi, S.; Khajavi, R.; Rashidi, A.; Rahimi, M.K.; Bahador, A. Novel PLA/ZnO Nanofibrous Nanocomposite Loaded with Tranexamic Acid as an Effective Wound Dressing: In Vitro and In Vivo Assessment. Iranian Journal of Biotechnology 2021, 19, 38–47, doi:10.30498/ijb.2021.220458.2737.
40. Qi, L.; Ou, K.; Hou, Y.; Yuan, P.; Yu, W.; Li, X.; Wang, B.; He, J.; Cui, S.; Chen, X. Unidirectional Water-Transport Antibacterial Trilayered Nanofiber-Based Wound Dressings Induced by Hydrophilic-Hydrophobic Gradient and Self-Pumping Effects. Materials & Design 2021, 201, 109461, doi:10.1016/j.matdes.2021.109461.
41. Charernsriwilaiwat, N.; Opanasopit, P.; Rojanarata, T.; Ngawhirunpat, T. Lysozyme-Loaded, Electrospun Chitosan-Based Nanofiber Mats for Wound Healing. International Journal of Pharmaceutics 2012, 427, 379–384, doi:10.1016/j.ijpharm.2012.02.010.
42. Heo, D.N.; Yang, D.H.; Lee, J.B.; Bae, M.S.; Kim, J.H.; Moon, S.H.; Chun, H.J.; Kim, C.H.; Lim, H.-N.; Kwon, I.K. Burn-Wound Healing Effect of Gelatin/Polyurethane Nanofiber Scaffold Containing Silver-Sulfadiazine. Journal of Biomedical Nanotechnology 2013, 9, 511–515, doi:10.1166/jbn.2013.1509.
43. Rujitanaroj, P.; Pimpha, N.; Supaphol, P. Wound-Dressing Materials with Antibacterial Activity from Electrospun Gelatin Fiber Mats Containing Silver Nanoparticles. Polymer 2008, 49, 4723–4732, doi:10.1016/j.polymer.2008.08.021.
44. Chen, J.-P.; Chiang, Y. Bioactive Electrospun Silver Nanoparticles-Containing Polyurethane Nanofibers as Wound Dressings. J Nanosci Nanotechnol 2010, 10, 7560–7564, doi:10.1166/jnn.2010.2829.
45. Xu, C.; Xu, F.; Wang, B.; Lu, T. Electrospinning of Poly(Ethylene-Co-Vinyl Alcohol) Nanofibres Encapsulated with Ag Nanoparticles for Skin Wound Healing. Journal of Nanomaterials 2011, 2011, 1–7, doi:10.1155/2011/201834.
46. Shalumon, K.T.; Anulekha, K.H.; Nair, S.V.; Nair, S.V.; Chennazhi, K.P.; Jayakumar, R. Sodium Alginate/Poly(Vinyl Alcohol)/Nano ZnO Composite Nanofibers for Antibacterial Wound Dressings. International Journal of Biological Macromolecules 2011, 49, 247–254, doi:10.1016/j.ijbiomac.2011.04.005.
47. Kim, K.; Luu, Y.K.; Chang, C.; Fang, D.; Hsiao, B.S.; Chu, B.; Hadjiargyrou, M. Incorporation and Controlled Release of a Hydrophilic Antibiotic Using Poly(Lactide-Co-Glycolide)-Based Electrospun Nanofibrous Scaffolds. Journal of Controlled Release 2004, 98, 47–56, doi:10.1016/j.jconrel.2004.04.009.
48. Monteiro, N.; Martins, M.; Martins, A.; Fonseca, N.A.; Moreira, J.N.; Reis, R.L.; Neves, N.M. Antibacterial Activity of Chitosan Nanofiber Meshes with Liposomes Immobilized Releasing Gentamicin. Acta Biomaterialia 2015, 18, 196–205, doi:10.1016/j.actbio.2015.02.018.
49. Jannesari, M.; Varshosaz, J.; Morshed, M.; Zamani, M. Composite Poly(Vinyl Alcohol)/Poly(Vinyl Acetate) Electrospun Nanofibrous Mats as a Novel Wound Dressing Matrix for Controlled Release of Drugs. Int J Nanomedicine 2011, 6, 993–1003, doi:10.2147/IJN.S17595.

50. Liu, X.; Lin, T.; Gao, Y.; Xu, Z.; Huang, C.; Yao, G.; Jiang, L.; Tang, Y.; Wang, X. Antimicrobial Electrospun Nanofibers of Cellulose Acetate and Polyester Urethane Composite for Wound Dressing. Journal of Biomedical Materials Research Part B: Applied Biomaterials 2012, 100B, 1556–1565, doi:10.1002/jbm.b.32724.
51. Thakur, R.A.; Florek, C.A.; Kohn, J.; Michniak, B.B. Electrospun Nanofibrous Polymeric Scaffold with Targeted Drug Release Profiles for Potential Application as Wound Dressing. International Journal of Pharmaceutics 2008, 364, 87–93, doi:10.1016/j.ijpharm.2008.07.033.
52. Merrell, J.G.; McLaughlin, S.W.; Tie, L.; Laurencin, C.T.; Chen, A.F.; Nair, L.S. Curcumin Loaded Poly(ε-Caprolactone) Nanofibers: Diabetic Wound Dressing with Antioxidant and Anti-Inflammatory Properties. Clin Exp Pharmacol Physiol 2009, 36, 1149–1156, doi:10.1111/j.1440-1681.2009.05216.x.
53. Xie, Z.; Paras, C.B.; Weng, H.; Punnakitikashem, P.; Su, L.-C.; Vu, K.; Tang, L.; Yang, J.; Nguyen, K.T. Dual Growth Factor Releasing Multi-Functional Nanofibers for Wound Healing. Acta Biomaterialia 2013, 9, 9351–9359, doi:10.1016/j.actbio.2013.07.030.
54. Lai, H.-J.; Kuan, C.-H.; Wu, H.-C.; Tsai, J.-C.; Chen, T.-M.; Hsieh, D.-J.; Wang, T.-W. Tailored Design of Electrospun Composite Nanofibers with Staged Release of Multiple Angiogenic Growth Factors for Chronic Wound Healing. Acta Biomaterialia 2014, 10, 4156–4166, doi:10.1016/j.actbio.2014.05.001.
55. Choi, J.S.; Leong, K.W.; Yoo, H.S. In Vivo Wound Healing of Diabetic Ulcers Using Electrospun Nanofibers Immobilized with Human Epidermal Growth Factor (EGF). Biomaterials 2008, 29, 587–596, doi:10.1016/j.biomaterials.2007.10.012.
56. Yang, Y.; Xia, T.; Zhi, W.; Wei, L.; Weng, J.; Zhang, C.; Li, X. Promotion of Skin Regeneration in Diabetic Rats by Electrospun Core-Sheath Fibers Loaded with Basic Fibroblast Growth Factor. Biomaterials 2011, 32, 4243–4254, doi:10.1016/j.biomaterials.2011.02.042.
